# Long-term outcome of low-energy extracorporeal shockwave therapy on gluteal tendinopathy documented by magnetic resonance imaging

**DOI:** 10.1371/journal.pone.0197460

**Published:** 2018-07-17

**Authors:** Kyoung-Ho Seo, Joon-Youn Lee, Kyungjae Yoon, Jong Geol Do, Hee-Jin Park, So-Yeon Lee, Young Sook Park, Yong-Taek Lee

**Affiliations:** 1 Department of Physical Medicine & Rehabilitation, Dongtan Sacred Heart Hospital, Hallym University College of Medicine, Hwaseong-si, Gyeonggi-do, Republic of Korea; 2 Department of Physical & Rehabilitation Medicine, Kangbuk Samsung Hospital, Sungkyunkwan University School of Medicine, Seoul, Republic of Korea; 3 Department of Radiology, Kangbuk Samsung Hospital, Sungkyunkwan University School of Medicine, Seoul, Republic of Korea; 4 Department of Physical & Rehabilitation Medicine, Samsung Changwon Hospital, Sungkyunkwan University School of Medicine, Changwon-si, Gyeongsangnam-do, Republic of Korea; Universite de Nantes, FRANCE

## Abstract

**Background:**

Previous outcome studies for extracorporeal shock wave therapy (ESWT) have included clinically diagnosed greater trochanteric pain syndrome (GTPS). The purpose of this study is to investigate outcome of ESWT on GTPS with gluteal tendinopathy documented by magnetic resonance imaging (MRI).

**Methods:**

Medical records of 38 consecutive patients were retrospectively reviewed, who underwent ESWT for GTPS with MRI-documented gluteal tendinopathy (> 6 months). ESWT was conducted (1/week) when the Roles-Maudsley score (RMS) showed “Poor” or “Fair” grade after conservative treatment until RMS had reached “Good” or “Excellent” grade (treatment success) or until 12 treatments had been applied. Numeric rating scale (NRS) and RMS were evaluated before, 1 week after (immediate follow-up) and mean 27 months after ESWT program (long-term follow-up). Success rate was calculated at each follow-up point.

**Results:**

Initial NRS (5.9 ± 1.6) significantly decreased at immediate (2.5 ± 1.5, *p*< 0.01) and long-term follow-up (3.3 ± 3.0, *p*< 0.01), respectively. Success rates were 83.3% (immediate) and 55.6% (long-term), respectively. There was no correlation among age, symptom duration and NRS.

**Conclusion:**

Low-energy ESWT can be an effective treatment for pain relief in chronic GTPS with MRI-documented gluteal tendinopathy. However, its long-term effect appears to decrease with time.

## Introduction

Greater trochanteric pain syndrome (GTPS) refers to a clinical condition with pain and tenderness at or around greater trochanter, which can radiate to the lateral aspect of the hip or thigh [1,2]. Classically, GTPS has been attributed to the trochanteric bursitis but more recent studies suggest that this condition involves degeneration, and/or tearing of the gluteal tendons. Although GTPS is usually diagnosed based on clinical findings such as history and physical examination, specific signs to diagnose this condition are not established. On the other hand, magnetic resonance imaging (MRI) is useful to demonstrate the pathology in and around greater trochanter or gluteal tendon as well as exclude other causes of lateral hip pain [3–6].

Primary management of GTPS is conventional conservative management including rest, non-steroid anti-inflammatory drug, physiotherapy, and corticosteroid injection. In refractory cases, operative treatment such as lengthening of iliotibial band and fascia lata may be considered [1,2,5]. Several studies reported extracorporeal shock wave therapy (ESWT) as a suitable alternative treatment option for refractory GTPS with satisfactory long-term maintenance [7–10]. However, these studies included clinically diagnosed GTPS. Therefore, subjects with various conditions could be included, which alters the outcome, because accurate clinical diagnosis of gluteal tendinopathy is always difficult.

To our knowledge, no study has investigated the outcome of ESWT on clinically diagnosed GTPS with MRI-documented gluteal tendinopathy. The aim of our study was to investigate the effect of ESWT for chronic refractory GTPS with gluteal tendinopathy documented by MRI.

## Materials and methods

### Subjects

**Fig 1:**
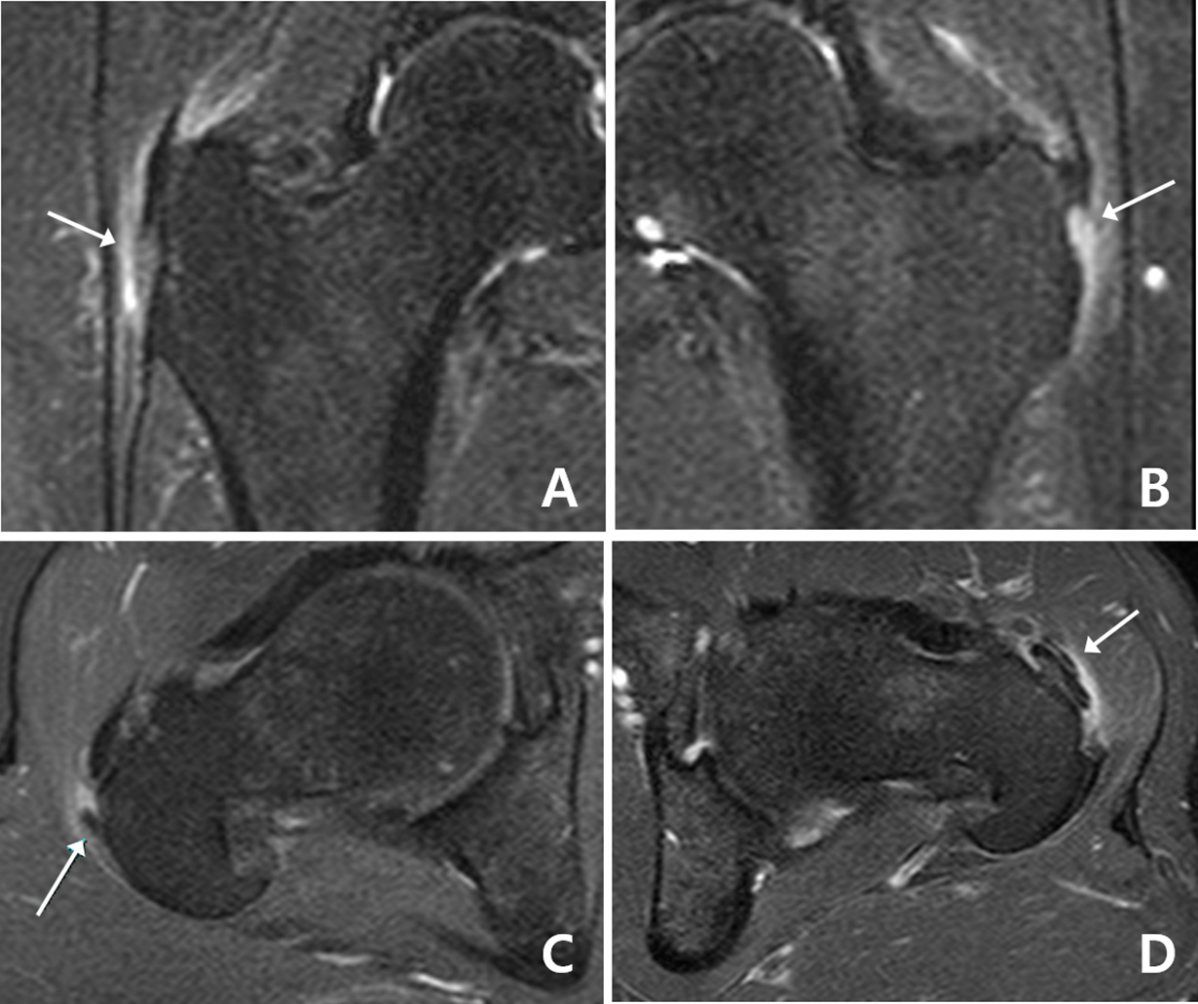
Findings of magnetic resonance imaging of gluteal tendinopathy. A: Mild increased intrasubstance signal and surrounding edema (arrow) suggests gluteus medius tendinosis with peritendinitis. B: Marked increased intrasubstance signal and ill-definition of the gluteus medius tendon near the insertion on the lateral facet (arrow) suggests insertional partial tear. C: Contrast-enhanced axial T1 with fat saturation MR images demonstrates a hypointense calcium deposit (arrow) in the gluteus medius tendon with surrounding edema. D: Mild increased intrasubstance signal and surrounding edema (arrow) suggests gluteus minimus tendinosis with peritendinitis.

**Fig 2:**
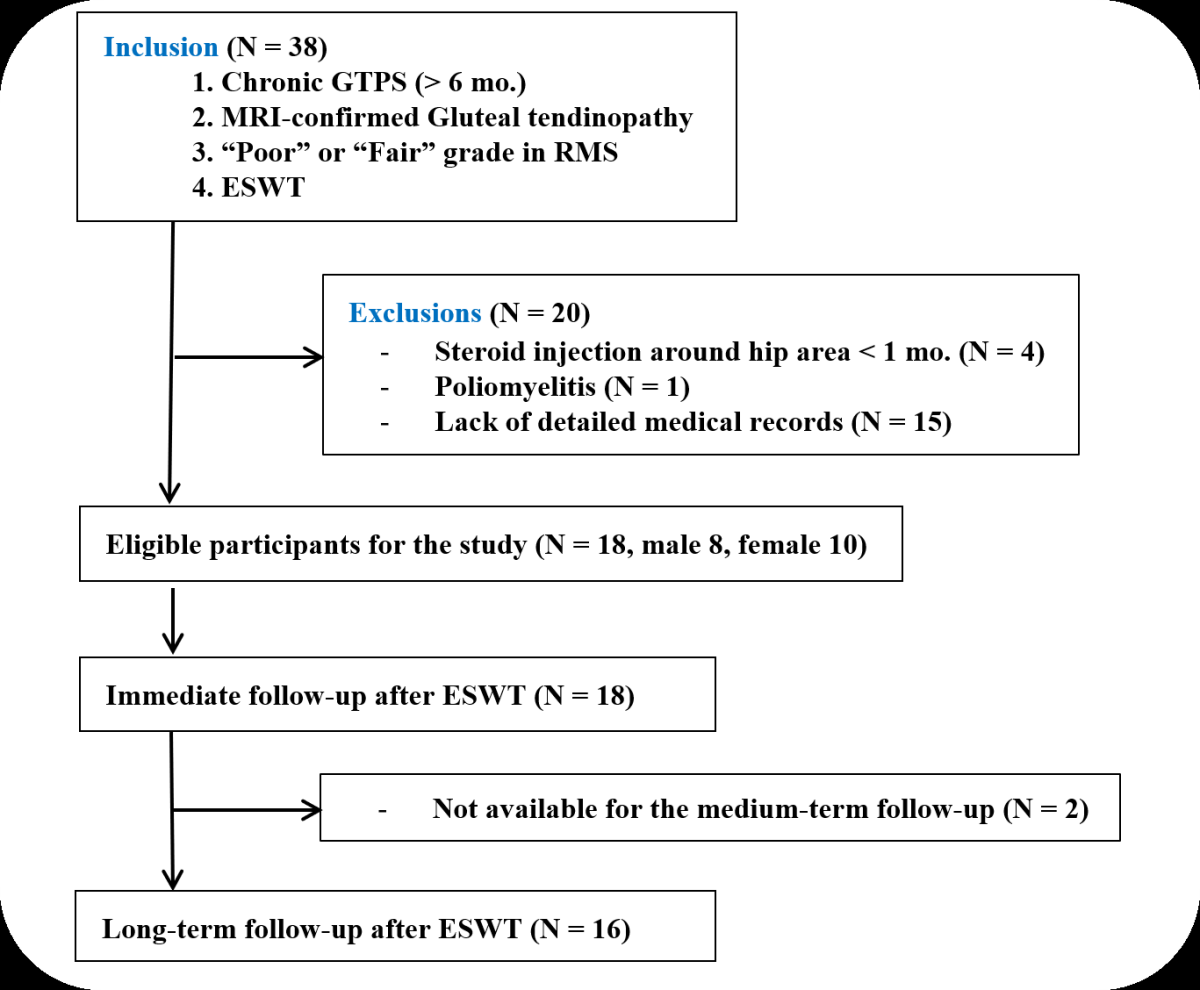
Flow chart for the inclusion of eligible subjects in this study.

We retrospectively reviewed the medical records of 38 consecutive patients who underwent ESWT with clinical diagnosis of chronic GTPS and MRI-confirmed gluteal tendinopathy. Chronic GTPS was diagnosed if patients had pain in the lateral aspect of hip or thigh impairing their daily activities for more than 6 months, tenderness over the greater trochanter and at least one positive finding on the clinical tests including lateral hip pain with a FABER test, resisted external derotation test, or single leg stance test. MRI-documented gluteal tendinopathy was determined when MRI showed abnormal findings in and around gluteal tendon as follow: tendinosis; peritendinitis; partial tear or calcific tendinitis (Fig 1). Exclusion criteria consisted of the following: 1) previous steroid injection around hip area within 1 months; 2) systemic inflammatory disease; 3) history of hip or spinal surgery; 4) lumbar radiculopathy; 5) hip joint arthritis finding on simple x-ray or MRI; 6) neurologic disorder of lower limb; and 7) complete rupture of gluteal tendon. Twenty patients were excluded for the following reasons: corticosteroid injection around hip area within 1 months in four (hip joint in 2, trochanteric bursa in 1, and ischial bursa in 1); poliomyelitis in 1; and lack of detailed medical records in 15. Finally, 18 patients who met our eligibility criteria were included (Fig 2). This study was approved by institutional ethics review board in Kangbuk Samsung Hospital, and the requirement for informed consent was waived because of the retrospective study design. (KBSMC 2016-09-018-001)

### Clinical assessment

Outcome measures consisted of the numerical rating scale (NRS), the Roles and Maudsley score (RMS) and treatment success rate. The NRS is an 11-point pain intensity rating scale, where 10 points indicated worst possible pain and 0 point no pain. The RMS is a subjective 4-point patient assessment of pain and limitations of activity (Table 1). NRS and RMS were assessed at baseline and 1 week after each ESWT. Immediate follow-up was conducted 1 week after ESWT program. Long-term follow-up was assessed at least 4 months (mean 27 months) after ESWT program by telephone interview. ESWT program was terminated when RMS had reached “Good” or “Excellent” grade, or when patient had requested stopping of ESWT due to lasting pain or increase of pain. Treatment success was determined when RMS reached “Good” or “Excellent”. Treatment failure was determined if RMS did not reach “Good” or “Excellent” when ESWT program was terminated, or corticosteroid injection was needed after ESWT program, or follow-up was missing.

**Table 1:** Roles and Maudsley score.

	Point	Interpretation
Excellent	1	No pain, full movement and activity
Good	2	Occasional discomfort, full movement and activity
Fair	3	Some discomfort after prolonged activity
Poor	4	Pain-limiting activities

### ESWT protocol

ESWT (0.10 mJ/mm^2^; 600 shocks per session) was conducted when patient’s symptom did not improved after previous conventional conservative treatment and still showed “Poor” or “Fair” grade in the Roles-Maudsley score (RMS) (Table 1). Patients underwent ESWT at intervals of 1 week until RMS had reached “Good” or “Excellent” grade (treatment success) or until 12 treatments had been applied. ESWT was applied using Evotron^Ⓡ^ (SwiTech, Kreuzlingen, Swizerland), the electrohydraulic type. While patient lied with lateral decubitus position, shockwave was applied to the area with maximal tenderness at or around greater trochanter. The probe with 25–45 mm of penetration depth was used. The frequency of shockwave was 1 Hz. Local anesthetics was not used.

### MRI evaluation

MRI examinations were performed using 1.5T MRI scanner (Signa; General Electric, Milwaukee, WI, USA) using a dedicated hip coil (Medical Advances, Milwaukee, WI, USA). MR images were evaluated by two radiologists in consensus on high-resolution monitors of a PACS.

### Statistical analysis

We used repeated measures ANOVA to analyze the immediate and long-term effect of ESWT on the subjective pain. Correlations between the following variables: age; symptom duration; and NRS (baseline and at each follow-up) were assessed by Pearson’s correlation coefficient. All analyses were performed with the Statistical Package for the Social Sciences software, Version 18.0 (SPSS, Chicago, Illinois, USA). *P* values <0.05 were considered statistically significant.

## Results

**Fig 3:**
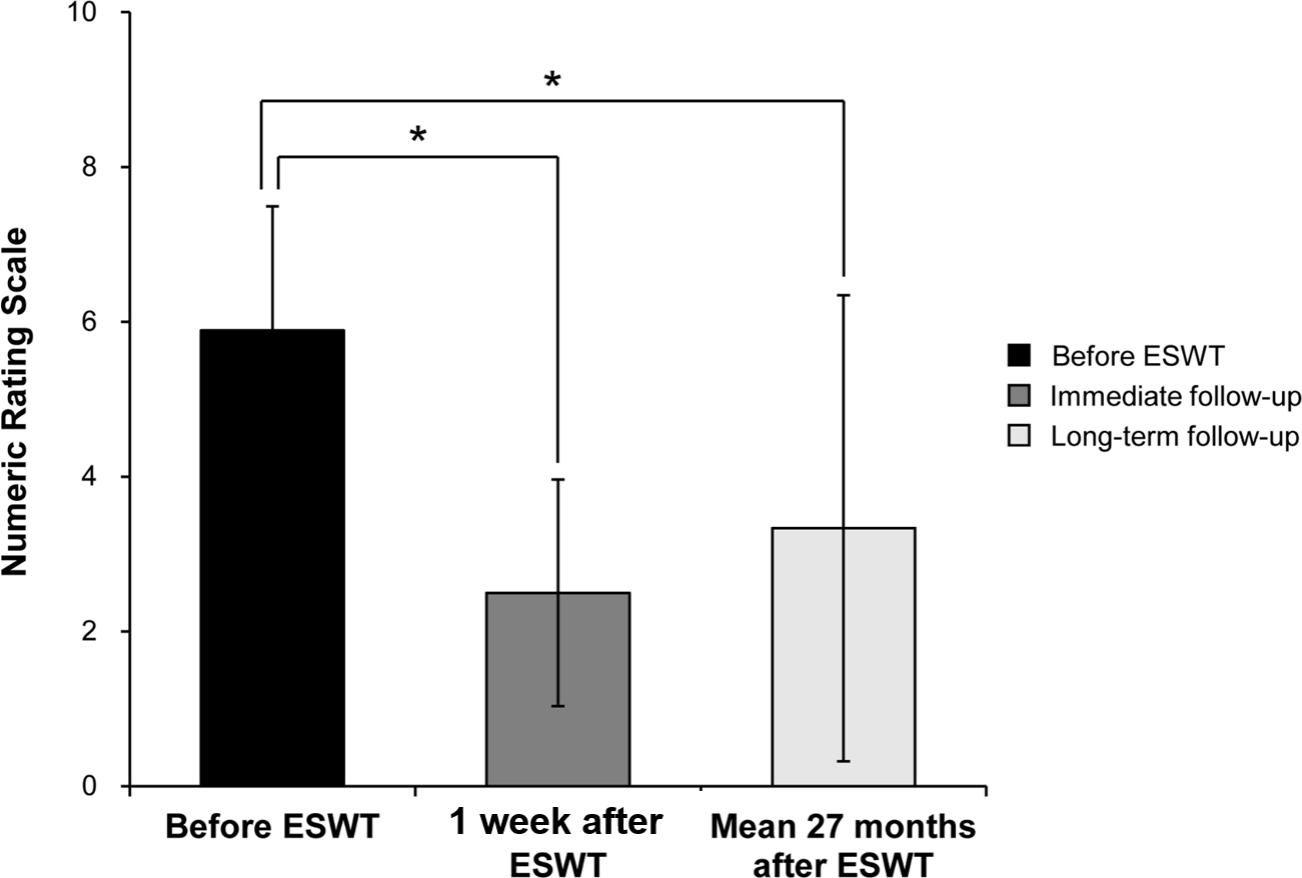
Outcome of ESWT for subjective pain at immediate follow-up (1 week after ESWT program) and long-term follow-up (mean 27 months after ESWT program).

Eighteen patients who met our eligibility criteria were included. The basic characteristics of the subjects were shown in Table 2. At long-term follow-up, two patients were not available for telephone interview and another two patients underwent corticosteroid injection after immediate follow-up. To avoid inflated outcomes, we replaced the missing RMS and NRS values of these 4 patients with the worst available assessment value, and also regarded as treatment failure (Table 2, S1 Dataset). Mean initial NRS before ESWT (5.9 ± 1.6) significantly decreased at immediate follow-up (2.5 ± 1.5, p< 0.01) and long-term follow up (3.3 ± 3.0, p< 0.01), respectively (Fig 3). However, NRS did not show significant difference between immediate and long-term follow-up while it showed tendency of increase with time (Fig 3). The overall success rates of ESWT for immediate and long-term follow-up were 83.3% and 55.6%, respectively. The Pearson correlation coefficients did not show correlation among age, symptom duration and NRS. Side effect of the ESWT such as bruising and swelling were not found in the present study.

**Table 2:** Demographic characteristics of subjects and outcome of ESWT.

No.	Sex	Age	symptom duration (months)	MRI findings	ESWT session	Initial		Immediate f/u		Long-term f/u	
						RMS	NRS	RMS	NRS	RMS	NRS
1	F	78	24^**a**^	Gmed tendinosis, peritendinitis	4	P	6	G	4	E	0
2	M	52	12	Gmed peritendinitis	6	P	7	F	6	F	5
3	M	48	24	Gmed partial tear, peritendinitis	4	F	7	G	2	INJ^**c**^	INJ^**c**^
4	M	58	24	Gmed tendinosis	11	P	4	G	2	G	2
5	F	71	12	Gmed tendinosis, peritendinitis	5	P	5	G	1	G	1
6	F	65	24	Gmin calcific tendinitis	1	P	6	E	0	E	0
7	M	72	7	Gmed calcific tendinitis	4	P	8	G	2	E	0
8	F	64	6	Gmed tendinosis	6	P	6	G	4	G	4
9	F	48	6^**b**^	Gmed calcific tendinitis	2	F	5	F	4	E	1
10	F	63	180	Gmed calcific tendinitis	10	F	3	G	1	G	1
11	M	53	60	Gmed tendinosis, peritendinitis	3	P	10	G	3	NA^**c**^	NA^**c**^
12	F	67	24	Gmed tendinosis	6	P	6	E	2	P	8
13	F	51	11	Gmin tendon partial tear	6	P	6	G	2	F	4
14	M	74	108	Gmed tendinosis	7	F	4	G	3	INJ^**c**^	INJ^**c**^
15	M	64	12	Gmed tendinosis	9	P	5	G	1	E	0
16	F	31	8	Gmed tendinosis	6	P	7	G	2	G	3
17	F	49	24^**a**^	Gmed tendinosis, peritendinitis	4	P	6	G	2	NA^**c**^	NA^**c**^
18	M	54	6^**b**^	Gmed calcific tendinitis	6	P	5	P	4	P	4

**ESWT**, extracorporeal shock wave therapy; **RMS**, Rolls-Maudsley score; **NRS**, numeric rating scale; **M**, male; **F**, female; **Gmed**, gluteus medius; **Gmin**, gluteus minimus; **P**, poor; **F**, fair; **G**, good; **E**, excellent; **INJ**, Corticosteroid injection after ESWT program; **NA**, Data was not available

**a**, Symptom duration was over 24 months but patients could not remember the exact onset time.; **b**, Symptom duration was over 6 months but patients could not remember the exact onset time; **c,** Missing RMS and NRS values were replaced with the worst available assessment value and regarded as treatment failure

## Discussion

The results of our study demonstrated that subjective pain started to decrease significantly at immediate follow-up (1 week after ESWT program). NRS did not show significant difference between immediate and long-term follow-up (mean 27 months after) while it showed tendency of increase with time (Fig 3). These results are similar with report of Furia et al. [9] that pain decreased significantly at one, three and twelve months after ESWT, respectively. Our findings are also supported by those of Rompe et al., who reported that ESWT was even more successful in the treatment of GTPS than corticosteroid injection at 4-month and 15-month follow-up although at one month from baseline, corticosteroid injection were better than ESWT [8].

Standard protocol of ESWT for GTPS is not yet established. Two reported well designed studies in the literature used different treatment protocol although both studies applied a standardized number of treatment sessions. Furia et al. [9] performed just a single session of ESWT (0.18 mJ/mm^2^; 2,000 shocks per session; total 360 mJ/mm^2^ per session) and Rompe et al. [8] conducted 3 sessions of ESWT (0.12 mJ/mm^2^, 2,000 shocks per session; total 240 mJ/mm^2^ per session). These two studies applied different total energy which is usually defined by EFD multiplied by total number of shocks applied. It has been reported that over-delivered shockwave energy is likely to be associated with greater pain and tissue damage, which may result in treatment failure [11,12]. In our daily practice, we try to avoid excessive delivery of total energy per each treatment session by using less number of shocks per session with more treatment sessions. In previous outcome study on chronic refractory Achilles tendinopathy, Lee et al. reported good long-term outcome with this protocol [13]. Therefore, in present study, modified protocol from previous study was used as follow: a maximum of 12 sessions of ESWT (0.10 mJ/mm^2^; 600 shocks per session; total 60 mJ/mm^2^ per session) until treatment success [13]. Consequently, total energy per each treatment session was much less (60 mJ/mm^2^) than Furia (360 mJ/mm^2^) and Rompe (240 mJ/mm^2^). In addition, the number of treatment session was inevitably flexible ranging from 1 to 11 sessions according to patient’s response, which makes it difficult to compare with former studies.

The present study is unique and differs from former reports because we first addressed the outcome of low-energy ESWT in chronic refractory GTPS with MRI-documented gluteal tendinopathy while previous studies included subjects with clinically diagnosed GTPS [7–9]. GTPS may be caused by impingement of the iliotibial band, tendinopathy, tendon tearing and inflammation of bursae [4,6]. This condition is difficult to diagnose clinically as it can resemble other musculoskeletal disorders, including hip joint conditions and lumbar spinal diseases and so on [9,14–17]. Furthermore, clinical diagnostic criteria for GTPS have not been universally established, which can create misdiagnosis and may result in erroneous conclusions regarding treatment effectiveness [15,16,18]. MRI is regarded as a useful tool for the diagnosis of gluteal tendinopathy and exclusion of other musculoskeletal disorders with pain at or around greater trochanter [3,5,6,19]. Previous studies that compared MRI with surgical pathologic findings demonstrated MRI is highly sensitive diagnostic investigation tool for GTPS [5,20,21]. Moreover, other studies reported that specificity of MRI for diagnosing trochanteric bursitis and gluteal tendon tears was found to be nearly 100% [22,23]. However, several researchers reported that although gluteal tendon pathology is considered important in defining GTPS, the findings of peritrochanteric edema and tendinosis on MRI are shown in many patients without GTPS symptoms [4,24]. Thus, correlation of clinical findings of GTPS with abnormal MRI findings would be very important to optimize the treatment strategies.

Previous studies demonstrated MRI appearances of gluteal tendinopathy as follows: soft tissue edema surrounding intact tendon (peritendinitis); thickening or increased intrasubstance T2 hyperintensity (tendinosis); focal absence of intact tendon fibers (partial tear); or tendon discontinuity (complete tear). [3,6,20] In our study, subjects did not have complete tear. They showed peritendinitis, tendinosis, calcific tendinitis, or partial tear in gluteus medius or minimus tendon on MRI.

Although ultrasonography is most commonly selected option for investigation of GTPS due to its easy accessibility and cost-effectiveness, sensitivity for the detection of low-grade tears is relatively low (61%). [22,25] In addition, ultrasonography has narrower field of view than MRI and difficulty to evaluate intraarticular structures, which make it difficult to figure out topographic anatomy around greater trochanter and rule out hip joint problems.

There are several limitations in this study that should be discussed. Firstly, small number of subjects with the exclusion of nearly 60% of the intended cohort is a source of bias that may reduce statistical power and alter the outcomes. Secondly, the lack of blinding as well as selection bias is likely associated with artificially inflated clinical outcomes. Thirdly, there was no control group. Thus, the effect of natural progression of the condition cannot be ruled out. Lastly, this study was conducted by retrospective chart review for immediate outcome and cross-sectional data gathering using telephone interview for long-term outcome. Therefore, there is no standardized outcome measure time point for long-term follow-up because all the telephone interviews on each subject were made at one point in time. Further studies are needed to supplement these limitations.

## Conclusions

When the diagnosis is established through meticulous physical examination and MRI, low-energy ESWT seems to be an effective treatment option for pain relief in chronic refractory GTPS. However, its long-term effect appears to decrease with time.

## Supporting information

S1 Dataset Dataset for characteristics of subjects and outcome of ESWT. (SAV)
